# Identification of Capsid/Coat Related Protein Folds and Their Utility for Virus Classification

**DOI:** 10.3389/fmicb.2017.00380

**Published:** 2017-03-10

**Authors:** Arshan Nasir, Gustavo Caetano-Anollés

**Affiliations:** ^1^Department of Crop Sciences, Evolutionary Bioinformatics Laboratory, University of Illinois at Urbana-ChampaignUrbana, IL, USA; ^2^Department of Biosciences, COMSATS Institute of Information TechnologyIslamabad, Pakistan

**Keywords:** capsid, virion, protein structure, virus taxonomy, SCOP, fold superfamily

## Abstract

The viral supergroup includes the entire collection of known and unknown viruses that roam our planet and infect life forms. The supergroup is remarkably diverse both in its genetics and morphology and has historically remained difficult to study and classify. The accumulation of protein structure data in the past few years now provides an excellent opportunity to re-examine the classification and evolution of viruses. Here we scan completely sequenced viral proteomes from all genome types and identify protein folds involved in the formation of viral capsids and virion architectures. Viruses encoding similar capsid/coat related folds were pooled into lineages, after benchmarking against published literature. Remarkably, the *in silico* exercise reproduced all previously described members of known structure-based viral lineages, along with several proposals for new additions, suggesting it could be a useful supplement to experimental approaches and to aid qualitative assessment of viral diversity in metagenome samples.

## Introduction

The last few years have dramatically increased our knowledge about viral systematics and evolution. The discoveries of “giant” viruses (La Scola et al., 2003; Arslan et al., 2011; Philippe et al., 2013; Legendre et al., 2014, 2015) and their virophages (La Scola et al., 2008; Desnues et al., 2012; Gaia et al., 2014; Levasseur et al., 2016) along with accumulation of large-scale protein structure and function data enabled testing hypotheses regarding the origin, classification, and evolution of the viral supergroup. This led to data-driven hypotheses of viral evolution (Koonin et al., 2006; Nasir and Caetano-Anollés, 2015) and new schemes for classifying viruses, different from traditional classification approaches that use genome features (Baltimore, 1971) or host/geographical preferences (King et al., 2012). For example, Bamford and coworkers proposed to define novel viral lineages based on the three-dimensional (3D) structural similarities of major viral capsid/coat proteins and virion assembly pathways (Abrescia et al., 2010). Under this classification, the many known viral families infecting distantly related hosts were pooled into four major viral lineages, the Picornavirus-like lineage, the PRD1/Adenovirus-like lineage, the HK97-like lineage, and the BTV-like lineage (Abrescia et al., 2012). These lineages were mainly described for *icosahedral* viruses however *helical* and *enveloped* viruses are also believed to fall into a limited number of lineages (Abrescia et al., 2012). Interestingly, member viruses of the PRD1/Adenovirus and HK97-like lineages infect species in all three domains of cellular life, Archaea, Bacteria, and Eukarya (Woese et al., 1990). Stark differences in membrane composition and cellular biology exist among cellular domains that likely hinder horizontal transfer of viruses between domains of life (Nasir et al., 2014, 2015). Thus, the structural and genetic similarities of viruses infecting the three cellular domains suggest they likely originated prior to the origin of modern diversified cells (Benson et al., 2004; Krupovič and Bamford, 2008). This scenario is also supported by our recent phylogenomic exploration of the origin of viral and cellular proteomes (Nasir and Caetano-Anollés, 2015). While the member viruses within a lineage exhibit strong 3D structural similarities in capsid/coat fold architectures (or principles in constructing a functional virion) regardless of the viral replicon (i.e., DNA or RNA) and/or infected host type, the lineages however are believed to be unrelated to each other indicating the polyphyletic origin of viruses (Bamford, 2003).

The conservation of protein structure over long evolutionary distances (Chothia and Lesk, 1986; Caetano-Anolles and Caetano-Anolles, 2003; Illergård et al., 2009; Abroi and Gough, 2011; Caetano-Anollés and Nasir, 2012; Lundin et al., 2012) forms the backbone of structure-based viral classification (Abrescia et al., 2010, 2012). This concept is especially applicable to viral capsid proteins as there is strong evolutionary pressure to maintain the overall morphology of the virus particle (Abrescia et al., 2012). Moreover, the capsid is the only feature that distinguishes plasmids, integrated viral genomes, and other “naked” genetic elements from *bona fide* viruses (Abrescia et al., 2012). For these reasons, the capsid has been termed the virus “self” (Bamford, 2003) and viruses have been referred to as “capsid-encoding organisms” (in comparison to ribosome-encoding cellular organisms) (Raoult and Forterre, 2008). The idea is strengthened by the fact that only a limited number of virion morphotypes may be considered geometrically and energetically favorable (Bamford et al., 2005). Indeed, a quick glance of the Structural Classification of Proteins (SCOP) database (Andreeva et al., 2008; Fox et al., 2014) reveals only 19 fold superfamilies (FSFs) corresponding to keywords “capsid” or “coat” (Table 1). Remarkably, these FSFs are either very rare or completely absent in cellular proteomes (Nasir and Caetano-Anollés, 2015). These observations identify the capsid as a reliable marker for improving or revising the current taxonomy of viruses. The availability of the SCOP database, a “gold standard” in the structural classification of proteins, and development of algorithms required to scan viral proteins against hidden Markov model (HMM) libraries of known protein structures (Gough et al., 2001; Gough and Chothia, 2002) now enable us to computationally detect the “type” of capsid fold present in viruses.

**Table 1:** **List of 27 capsid/coat related FSFs as identified from SCOP (*E* < 0.0001), literature (Abrescia et al., 2012; Nasir and Caetano-Anollés, 2015), or keyword searches**.

**SCOP Id**	**SCOP *ccs***	**FSF Description**	**Lineage**	**Evidence**	**%A**	**%B**	**%E**
48345	a.115.1	A virus capsid protein alpha-helical domain	BTV-like lineage	Keyword	0.00	0.00	0.00
64465	d.196.1	Outer capsid protein sigma 3	BTV-like lineage	Keyword	0.00	0.09	0.00
82856	e.42.1	L-A virus major coat protein	BTV-like lineage	Keyword	0.00	0.00	1.04
49818	b.19.1	Viral protein domain	BTV-like lineage	Literature	0.00	0.00	0.00
56831	e.28.1	Reovirus inner layer core protein p3	BTV-like lineage	Literature	0.00	0.18	0.26
**58176**	**i.7.1**	**Reovirus components**	**BTV-like lineage**	**Literature**	**NA**	**NA**	**NA**
51274	b.85.2	Head decoration protein D (gpD, major capsid protein D)	HK97-like lineage	Keyword	0.00	0.72	0.00
56563	d.183.1	Major capsid protein gp5	HK97-like lineage	Keyword	9.84	32.74	1.04
103417	e.48.1	Major capsid protein VP5	HK97-like lineage	Keyword	0.00	0.00	0.26
**64612**	**i.14.1**	**Bacteriophage HK97 procapsid (prohead II)**	**HK97-like lineage**	**Keyword**	**NA**	**NA**	**NA**
48045	a.84.1	Scaffolding protein gpD of bacteriophage procapsid	Picornavirus-like lineage	Keyword	0.00	0.00	0.00
88633	b.121.4	Positive stranded ssRNA viruses	Picornavirus-like lineage	Literature	0.00	1.08	12.27
88645	b.121.5	ssDNA viruses	Picornavirus-like lineage	SCOP relative	0.00	0.00	4.18
88648	b.121.6	Group I dsDNA viruses	Picornavirus-like lineage	SCOP relative	0.00	0.00	0.00
88650	b.121.7	Satellite viruses	Picornavirus-like lineage	SCOP relative	0.00	0.00	0.00
49749	b.121.2	Group II dsDNA viruses VP	PRD1/Adenovirus-like lineage	SCOP relative	0.00	0.00	1.31
47353	a.28.3	Retrovirus capsid dimerization domain-like	Retrotranscribing-like lineage?	Keyword	0.00	0.00	17.23
47852	a.62.1	Hepatitis B viral capsid (hbcag)	Retrotranscribing-like lineage?	Keyword	0.00	0.00	0.00
47943	a.73.1	Retrovirus capsid protein, N-terminal core domain	Retrotranscribing-like lineage?	Keyword	0.00	0.00	5.22
50176	b.37.1	N-terminal domains of the minor coat protein g3p	Inovirus-like lineage?	Keyword	0.00	0.00	0.00
57987	h.1.4	Inovirus (filamentous phage) major coat protein	Inovirus-like lineage?	Keyword	0.00	0.99	0.00
103068	d.254.1	Nucleocapsid protein dimerization domain	*Nidovirales*-like lineage?	Keyword	0.00	0.00	0.00
55405	d.85.1	RNA bacteriophage capsid protein	*Leviviridae*-like lineage?	Keyword	0.00	0.00	0.26
101257	a.190.1	Flavivirus capsid protein C	Other/Unclassified	Keyword	0.00	0.00	0.00
47195	a.24.5	TMV-like viral coat proteins	Other/Unclassified	Keyword	0.00	0.00	4.18
**58668**	**j.54.1**	**Hepatitis C virus N-terminal capsid protein fragment 2-45**	**Other/Unclassified**	**Keyword**	**NA**	**NA**	**NA**
**118396**	**j.9.7**	**Ilarvirus coat protein N-terminal fragment**	**Other/Unclassified**	**Keyword**	**NA**	**NA**	**NA**

*Where available (23 out of 27), the distribution (%) in the proteomes of 122 Archaea (A), 1,115 Bacteria (B), and 383 Eukarya (E) are also given along with assignment to one of the four experimentally defined lineages (Abrescia et al., 2012) or to novel or “Other/Unclassified” category (see text). FSFs highlighted in bold were not detected in any of the studied 3,460 viral proteomes in Nasir and Caetano-Anollés (2015)*.

Here we survey capsid/coat related FSFs in the proteomes of 3,460 completely-sequenced viruses (corresponding to all seven known replicon types) with the broad objectives of characterizing each known viral lineage (benchmarked against Abrescia et al., 2012) and suggesting novel members for existing lineages (or even novel lineages). Remarkably, our computational exercise recovered the previously experimentally defined viral lineages along with proposals for new additions, suggesting it could be a reliable supplement to experimental approaches for rapid identification of viral lineages, for example, in metagenomic samples. Accurate assignment of viruses into known lineages will be especially invaluable for novel viruses for which little is known and experimental characterization is technically challenging. Importantly, and despite the great genetic diversity and host biases observed among modern viruses (Nasir et al., 2014; Koonin et al., 2015), virion construction principles appear generally and relatively more conserved in evolution than viral gene sequences or host-associated preferences and present a more viable classification approach for modern viruses (Bamford et al., 2005; Krupovič and Bamford, 2010, 2011), in addition to providing insights about viral origins and evolution (Nasir and Caetano-Anollés, 2015; Forterre, 2016).

## Material and methods

### Assignment of capsid/coat related FSFs

Capsid/coat related FSFs were first extracted from SCOP ver. 2.05 (last updated February 2015) using keywords “capsid” and “coat.” This yielded 14 capsid and 5 coat related FSFs (Table 1). Because, keyword search is directly dependent on how FSFs are described in SCOP (e.g., procapsid), this likely missed several genuine capsid/coat related FSFs. Therefore, we mapped the 17 Protein Data Bank (PDB) codes corresponding to the four experimentally-defined viral lineages (Abrescia et al., 2012) to SCOP 2.05 to get their FSF descriptions. Four PDB entries were not present in SCOP 2.05 (1YUE, 3C5B, 2BBD, and 2VVF) and thus were not considered. The remaining 13 PDB entries corresponded to 8 new FSFs, out of which four (b.121.4, b.19.1, e.28.1, and i.7.1) were new additions to the list (i.e., were not detected earlier by keyword search). The list was further refined by looking for SCOP relatives for each FSF (i.e., other FSFs part of the same fold). As a result, four more FSFs b.121.2, b.121.5, b.121.6, and b.121.7 were added to the list, as SCOP relatives of b.121.4. The final list included 27 FSFs (19 keywords, 4 SCOP relatives, and 4 from Abrescia et al., 2012), out of which 23 were detected in our sampled viral and cellular proteomes (highlighted in boldface in Table 1). Throughout the manuscript, FSFs are named using SCOP *concise classification strings* (*ccs*) for quick identification. For example, b.121.4 FSF belongs to SCOP class “b” (i.e., all-beta proteins), fold no. 121, and FSF no. 4 in that fold and class.

### Proteome data retrieval

Viral and cellular proteome data and FSF assignments were taken from Nasir and Caetano-Anollés (2015). FSF information was available for 3,460 viruses belonging to 1,649 dsDNA, 534 ssDNA, 166 dsRNA, 881 plus-ssRNA, 110 minus-ssRNA, 56 ssRNA-RT, and 64 dsDNA-RT viruses and 1,620 cellular organisms belonging to 122 Archaea, 1,115 Bacteria, and 383 Eukarya. Viral and cellular proteomes that gave a significant hit (*E* < 0.0001) to any of the 27 capsid/coat related FSFs (Table 1) were kept for taxonomic assignment and manual inspection.

### Retrieval of virion-related proteins

A total of 6,478 manually curated and verified proteins tagged to “Virion” keyword in UniProtKB keywords category “Cellular component” were downloaded from http://www.uniprot.org/keywords/ (November 15, 2015). These proteins corresponded to all known viral replicons including dsDNA (*n* = 2,220), ssDNA (178), dsRNA (502), plus-ssRNA, (912), minus-ssRNA (1,849), dsDNA-RT (139), ssRNA-RT (629), and in addition, satellite viruses (6), unclassified virophages, phages, and viruses (9), and deltaviruses (34). These proteins were scanned against SUPERFAMILY HMMs (Gough et al., 2001; Gough and Chothia, 2002) for recognition of FSF domains using a stringent *E*-value cutoff < 0.0001.

## Results

We examined how the known capsid/coat related FSFs, identified via SCOP or from literature, corresponded to experimentally defined viral lineages (Abrescia et al., 2012) and examined their distribution in the 3,460 viral (corresponding to seven viral replicons) and 1,420 cellular (Archaea, Bacteria, and Eukarya) proteomes.

### The picornavirus-like lineage

**Figure 1:**
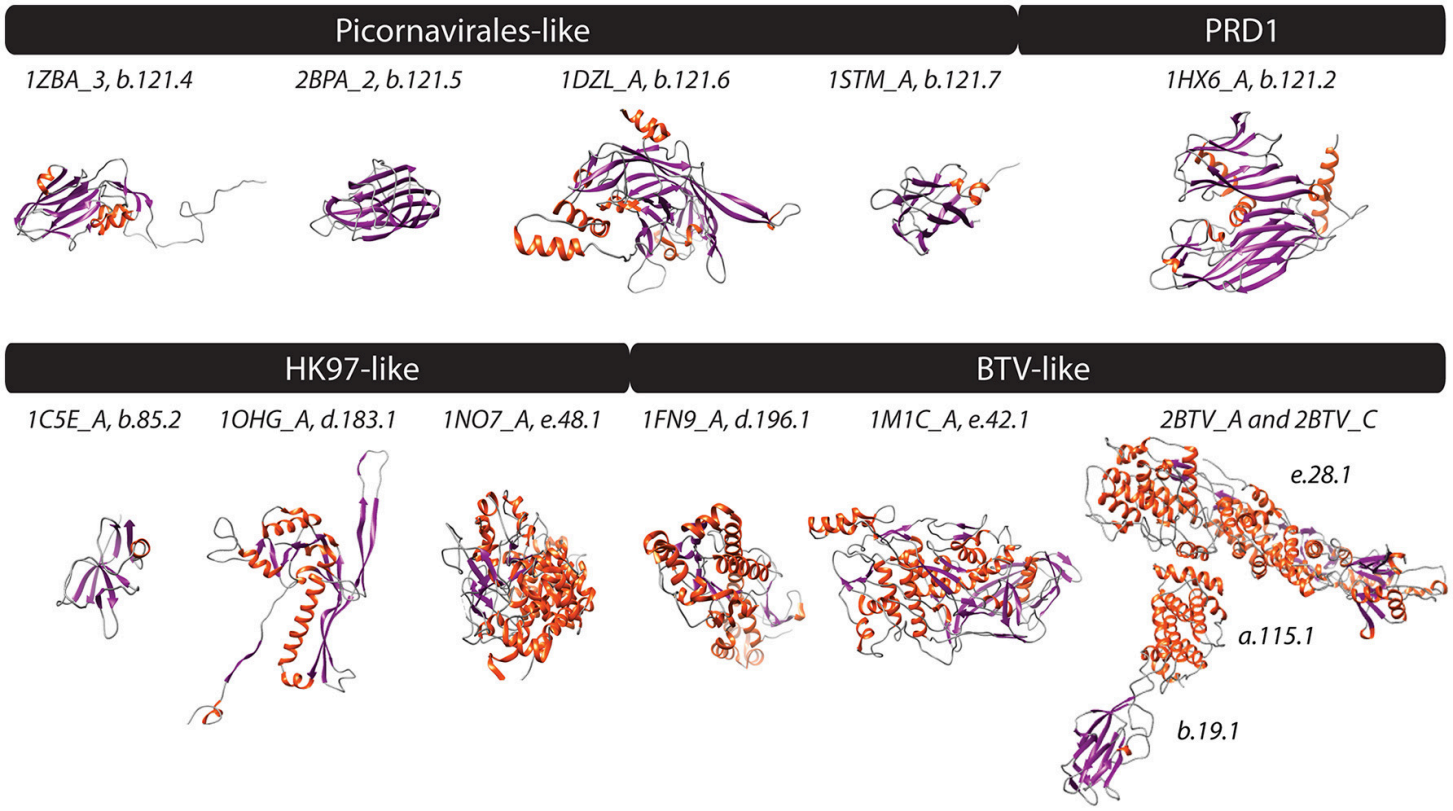
**PDB structures corresponding to FSFs of four experimentally defined viral lineages (Abrescia et al., 2012) are shown**. Helices, strands, and coils are colored red, blue, and gray, respectively. Text above structures indicates PDB ID along with chain information and FSF *ccs*. Additional monomers, ligands, and extra molecules were removed from visualization. 1ZBA, Foot-and-mouth disease virus; 2BPA, Bacteriophage phi-X174; 1DZL, Human papillomavirus type 16; 1STM, Satellite panicum mosaic virus; 1HX6, Bacteriophage PRD1; 1C5E, Bacteriophage lambda; 1OHG, Bacteriophage HK97; 1NO7, Herpes simplex virus 1; 1FN9, Reovirus; 1M1C, *Saccharomyces cerevisiae* virus L-A; and 2BTV, Bluetongue virus.

The Picornavirus-like lineage is characterized by the “jelly-roll” or “β-barrel” fold, which is commonly seen in RNA viruses (Abrescia et al., 2012). It is the largest defined viral lineage, including members from plus-ssRNA (*Bromoviridae, Caliciviridae, Comoviridae, Dicistroviridae, Luteoviridae, Nodaviridae, Picornaviridae, Sequiviridae, Tetraviridae, Tombusviridae, Tymoviridae*), dsRNA (*Birnaviridae*), ssDNA (*Microviridae, Parvoviridae*), and dsDNA (*Papillomaviridae*, and *Polyomaviridae*) viruses but no minus-ssRNA and retrotranscribing viruses, according to (Abrescia et al., 2012). Of these, *Comoviridae* and *Sequiviridae* are now classified under *Secoviridae*, which constitutes one of the five families in the viral order *Picornavirales* (other families being *Dicistroviridae, Iflaviridae, Marnaviridae*, and *Picornaviridae*). The “jelly-roll” fold has a topology of eight β-strands organized into two antiparallel sheets and is represented by the “Nucleoplasmin-like VP (viral coat and capsid proteins)” fold (b.121.1) in SCOP. The b.121 fold in the SCOP hierarchy includes 7 children FSFs (that are not necessarily related in evolution according to SCOP definitions): (i) “PHM/PNGase F” FSF (b.121.1) involved in oxidation-reduction metabolic processes (not detected in any of our sampled viral proteomes), (ii) “Group II dsDNA viruses VP” FSF (b.121.2), which is the “double β-barrel” fold signature of the PRD1/Adenovirus-like lineage (read below), (iii) “Nucleoplasmin-like core domain” FSF (b.121.3) involved in the assembly of nucleosomes in cells, and (iv-vii) FSFs b.121.4, b.121.5, b.121.6, and b.121.7 (Figure 1) that define the picornavirus-like lineage and are individually described below.

**Figure 2:**
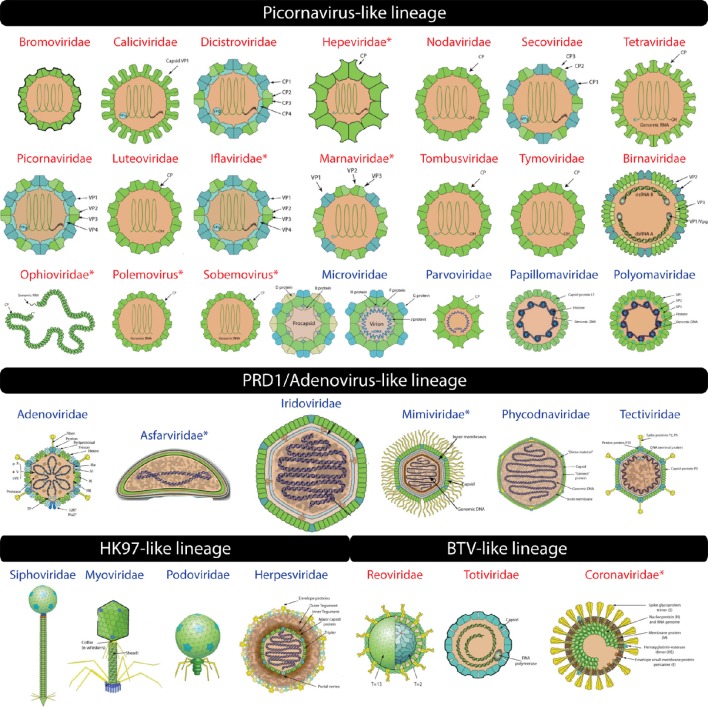
**Virion images of viral families representatives of the four experimentally defined lineages (Abrescia et al., 2012) are shown**. RNA and DNA viruses are shown in red and blue, respectively. Novel additions to existing lineages are indicated by an asterisk. Virion pictures were taken from ViralZone (Hulo et al., 2011) with permission from the Swiss Institute of Bioinformatics (SIB).

The “Positive stranded ssRNA viruses” FSF (b.121.4) was detected mostly in RNA viruses including plus-ssRNA (10 families), dsRNA (*Birnaviridae*), and the novel addition of minus-ssRNA (*Ophioviridae*) viruses (Table 2, Figure 2 for virion morphotypes). Thus, our computational approach extended the picornavirus-like lineage to also include minus-ssRNA viruses. Experimental work will be required to confirm if these viruses truly belong to this lineage. Other novel additions included polemoviruses and sobemoviruses (plus-ssRNA viruses that are yet to be assigned to a viral family), eight unclassified plus-ssRNA viruses, *Hepeviridae* (family of plus-ssRNA viruses that includes the human and avian hepatitis E viruses), *Iflaviridae* and *Marnaviridae* (thus completing the detection of all five *Picornavirales* families in our computational assignments) and one dsDNA virus belonging to *Myoviridae* (*Prochlorococcus phage P-SSM2*). Interestingly, *Myoviridae* possess the so-called “HK97” capsid/coat related fold also seen in eukaryotic *Herpesviridae*. Together they constitute the HK97-like lineage (read below) and are believed to be unrelated to the picornavirus-like lineage. Thus, assignment of FSF b.121.4 to *Myoviridae* could either be a false hit or suggests that the two lineages could (in fact) be distantly related. For example, unlike other dsRNA viruses that constitute the BTV-like lineage (read below), *Birnaviridae* share genomic (Birghan et al., 2000) and structural similarities (Coulibaly et al., 2005) with plus-ssRNA viruses. Based on our assignments, *Birnaviridae* fall into the picornavirus-like lineage and possess the b.121.4 FSF hallmark of plus-ssRNA viruses. However, the arrangement of the major capsid protein in birnaviruses is similar to the other members of the BTV-like lineage (Abrescia et al., 2012) casting doubts on its accurate affiliation. Perhaps, mixing of ancestral viruses of the picronavirus-like and BTV-like lineages led to modern birnaviruses (Coulibaly et al., 2005) or alternatively represent the evolutionary link between the two lineages.

**Table 2:** **Genome type, host range, taxonomy assignment, and member families are listed for 23 capsid/coat related FSFs detected in our sampled proteomes**.

**SCOP *ccs***	**Member families**	**Replicon**	**Host range**
**BTV-LIKE LINEAGE (5 FSFS)**			
a.115.1	*Reoviridae*	dsRNA	Algae, Fungi, Plants, Vertebrates, Invertebrates
d.196.1	*Reoviridae*	dsRNA	Algae, Fungi, Plants, Vertebrates, Invertebrates
e.42.1	*Totiviridae*	dsRNA	Fungi, Protozoa, Invertebrates, Vertebrates
b.19.1	*Reoviridae, Orthomyxoviridae, Coronaviridae*	dsRNA, plus-ssRNA, minus-ssRNA	Algae, Fungi, Plants, Vertebrates, Invertebrates
e.28.1	*Reoviridae*	dsRNA	Algae, Fungi, Plants, Vertebrates, Invertebrates
**HK97-LIKE LINEAGE (3 FSFS)**			
b.85.2	*Siphoviridae* and unclassified *Caudovirales*	dsDNA	Archaea, Bacteria
d.183.1	*Caudovirales*	dsDNA	Archaea, Bacteria
e.48.1	*Herpesviridae*	dsDNA	Vertebrates
**PICORNAVIRUS-LIKE LINEAGE (5 FSFS)**			
a.84.1	*Microviridae*	ssDNA	Bacteria
b.121.4	*Bromoviridae, Caliciviridae, Dicistroviridae, Hepeviridae, Nodaviridae, Secoviridae, Tetraviridae, Luteoviridae, Picornaviridae, Iflaviridae, Marnaviridae, Tombusviridae, Tymoviridae, Birnaviridae, Ophioviridae, Myoviridae, Poleomoviruses, Sobemoviruses* and unclassfied plus-ssRNA viruses	plus-ssRNA, dsRNA, minus-ssRNA, and dsDNA	Algae, Plants, Vertebrates, Invertebrates, Archaea, Bacteria
b.121.5	*Microviridae, Parvoviridae*, unclassified ssDNA virus	ssDNA	Bacteria, Vertebrates, Invertebrates
b.121.6	*Papillomaviridae, Polyomaviridae*	dsDNA	Vertebrates
b.121.7	Unclassified	ssDNA	Unknown
**PRD1/ADENOVIRUS-LIKE LINEAGE (1 FSF)**			
b.121.2	*Adenoviridae, Asco/Asfarviridae, Iridoviridae, Marsielleviridae, Mimiviridae, Phycodnaviridae, Tectiviridae*, Unclassified	dsDNA	Vertebrates, Invertebrates, Protozoa, Algae, Bacteria
**CANDIDATE RETROTRANSCRIBING-LIKE LINEAGE (3 FSFS)**			
a.28.3	*Retroviridae*	ssRNA-RT	Vertebrates
a.62.1	*Hepadnaviridae*	dsDNA-RT	Vertebrates
a.73.1	*Retroviridae*	ssRNA-RT	Vertebrates
**CANDIDATE INOVIRIDAE-LIKE LINEAGE (2 FSFS)**			
b.37.1	*Inoviridae*	ssDNA	Bacteria
h.1.4	*Inoviridae*	ssDNA	Bacteria
**CANDIDATE NIDOVIRALES-LIKE LINEAGE (1 FSF)**			
d.254.1	*Arteriviridae, Coronaviridae*	plus-ssRNA	Vertebrates
**CANDIDATE LEVIVIRIDAE-LIKE LINEAGE (1 FSF)**			
d.85.1	*Leviviridae*	plus-ssRNA	Bacteria
**OTHER/UNCLASSIFIED (2 FSF)S**			
a.190.1	Flaviviridae	plus-ssRNA	Vertebrates, Invertebrates,
a.24.5	*Benyviridae, Potyviridae, Virgaviridae*	plus-ssRNA	Plants

*Virus families hosts, as described by the NCBI Viral Genomes Resource (Bao et al., 2004)*.

*The Bao et al. (2004) paper has been provided in complete citation in the reference list at the appropriate position*.

The “ssDNA viruses” FSF (b.121.5) was detected in many ssDNA viruses of the *Microviridae* and *Parvoviridae* families. The capsid and spike proteins (F and G) of *Bacteriophage phiX174* (*Microviridae*) possess the “jelly-roll” fold and were reliably matched to b.121.5 (Figure 2). In addition, b.121.5 was also detected in an unclassified ssDNA virus *Dragonfly-associated microphage 1* possibly linking this virus to the lineage. *Microviridae* also possess another capsid/coat related FSF “Scaffolding protein gpD of bacteriophage procapsid” (a.84.1) that acts as a molecular chaperone and becomes part of the external scaffold of viral procapsid, which is later removed to release the mature virion (Figure 2, Dokland et al., 1997). Although a.84.1 is not part of the mature virion, it was uniquely detected in *Microviridae* and thus could still serve as marker to identify *Micorviridae* members together with b.121.5.

The “Group I dsDNA viruses” FSF (b.121.6) includes coat and L1 proteins from polyomaviruses and papillomaviruses, both established members of the picornavirus-like lineage. Finally, the “Satellite viruses” FSF (b.121.7) was detected in the *Circovirus-like genome RW_B* virus (ssDNA). It seems that the coat protein of this virus resembles the “jelly-roll” coat proteins of satellite viruses (e.g., *Satellite panicum mosaic virus*), which harbor a typical “jelly-roll” fold but with up to 1–2 additional β-strands (Ban et al., 1995). Thus, this FSF could be considered another specialized form of the “jelly-roll” fold.

Based on our computational assignments, the b.121.4, b.121.5, b.121.6, b.121.7 (and possibly a.84.1) FSFs can be used as candidates to recruit new members of the picornavirus-like lineage. The other members of the b.121 fold either include proteins specific to cells (i.e., b.121.1 and b.121.3) or advanced forms of the “jelly-roll” (b.121.2) that make a lineage of their own (read below). Importantly, the picornavirus-like lineage now includes viruses with all replicon types except two groups of retrotranscribing viruses and supports the idea that viruses with different replicons can share strong structural and molecular properties (Bamford, 2003). The exercise also revealed that structural relatives of the “jelly-roll” fold are found in cells (e.g., histone chaperones and metabolic folds Dutta et al., 2001; Liu et al., 2002; Cheng and Brooks, 2013) and thus it may not be a unique viral hallmark. However, none of the five putative picornavirus-like lineage associated FSFs (a.84.1, b.121.4, b.121.5, b.121.6, and b.121.7) were detected in any of the archaeal proteomes while b.121.4 was detected in roughly 1% of bacterial and 12% of eukaryotic proteomes, and b.121.6 in only 4% of eukaryotic proteomes indicating rare presence in cellular proteomes (Table 1). These rare occurrences could (possibly) be episodes of virus-to-cell horizontal gene transfer (HGT) during infection (Akita et al., 2007; Sutter et al., 2008). Indeed, b.121.4, a hallmark of plus-sense RNA viruses, was relatively widespread among eukaryotes (12% spread) consistent with previous knowledge that RNA viral infections are common in eukaryotic species but absent in Archaea and extremely rare in Bacteria (Nasir et al., 2014, 2015; Koonin et al., 2015). Importantly, the host range of the picornavirus-like lineage is apparently restricted to Bacteria and Eukarya (not accounting for Myoviridae that also infects Archaea) and 100% of the viral families listed in (Abrescia et al., 2012) were detected along with several new novel additions indicating the success of our computational survey.

### The PRD1/adenovirus lineage

The PRD1/Adenovirus-like lineage includes dsDNA viruses that infect species in the three cellular domains of life. The prototype members include the human adenoviruses (*Adenoviridae*), *Paramecium bursaria chlorella* viruses (*Phycodnaviridae*), the *Bacteriophage PRD1* (*Tectiviridae*), and the archaeal *Sulfolobus turreted icosahedral virus* (*Turriviridae*). The lineage is characterized by the “double jelly-roll” fold, which likely formed by the duplication of the “jelly-roll” fold (Krupovič and Bamford, 2011). However, the “jelly-roll” and “double jelly-roll” folds are utilized differently in assembling capsids and hence form two distinct lineages (Krupovič and Bamford, 2011). Capsids of viruses belonging to the PRD1/Adenovirus lineage are assembled in trimers consisting of two β-barrels arranged around a pseudo six-fold axis. The “double β-barrel” fold corresponds to “Group II dsDNA viruses VP” FSF (b.121.2) (Figure 1) and was detected in *Adenoviridae, Asco/Asfarviridae, Iridoviridae, Marseilleviridae, Mimiviridae, Phycodnaviridae, Tectiviridae*, and two unclassified dsDNA viruses (*Micromonas pusilla virus 12T* and *Ostreococcus lucimarinus virus OlV5*). Notable exceptions from (Abrescia et al., 2012) were of *Poxviridae, Corticoviridae*, and *Turriviridae*. However, the “double β-barrel” protein domain in poxviruses only facilitates virion formation and does not become part of the capsid. In turn, the corresponding PDB entries (2BBD and 2VVF) for *Turriviridae* and *Corticoviridae* (identified from Abrescia et al., 2012) were not part of SCOP and were thus missed by SCOP-based SUPERFAMILY HMMs (Gough et al., 2001; Gough and Chothia, 2002). Thus, their absence is likely not due to failure of our approach but due to incomplete coverage of PDB in SCOP. New additions of *Asco*/*Asfarviridae* and *Mimiviridae* were confirmed independently (Krupovič and Bamford, 2011). The “double β-barrel” is apparently a virus hallmark and was detected in only 1% of eukaryotic proteomes (Table 1), suggesting it was likely acquired in the few cellular proteomes from their viruses by HGT.

### The HK97-like lineage

The HK97-like lineage includes tailed viruses belonging to archaeal and bacterial *Caudovirales* (*Myoviridae, Podoviridae*, and *Siphoviridae*) and the eukaryotic *Herpesviridae* (Figure 2). The HK97 fold corresponds to two major FSFs, the “Major capsid protein gp5” (d.183.1) from *Bacteriophage HK97* and the “Major capsid protein VP5” (e.48.1) from *Herpes simplex virus 1* (Figure 1). It has been verified that the “floor” domain of herpesvirus VP5 and HK97 gp5 have similar structural organization and are evolutionarily related (Baker et al., 2005). Moreover, a small tail similar to that of *Podoviridae* has been detected in the herpesvirus capsid, further supporting their inclusion in the HK97-like lineage (Schmid et al., 2012). In addition, the “Head decoration protein D” (gpD, major capsid protein D) FSF (b.85.2) was also detected exclusively in *Siphoviridae* and one unclassified *Caudovirales* (*Providencia phage Redjac*). The b.85.2 is a “beta-clip” fold that forms an incomplete barrel somewhat similar to the “jelly-roll” structure (Figure 1). Its main function is to decorate the head shell and stabilize the capsid (Yang et al., 2000). There were no additional SCOP relatives for either d.183.1 or e.48.1, the two major markers for the HK97-like lineage. However, b.85.2 had six SCOP relatives: (i) “AFP III-like domain” (b.85.1), a type of antifreezing protein possessing a compact fold composed of beta-strands (Davies et al., 2002) (found in cells but not detected in any virus) (ii) “Urease, beta-subunit” (b.85.3), a subunit of urease enzyme known to hydrolyze urea into carbon dioxide and ammonia (Takishima et al., 1988) (again detected in cells but not in any virus) (iii) “dUTPase-like” (b.85.4), a metabolic enzyme near-universal in cells and also detected in a wide array of viruses, (iv) “Tlp20, baculovirus telokin-like protein” (b.85.5), a virus-specific protein of unknown function expressed late in baculoviruses (Raynes et al., 1994), (v) “MoeA C-terminal domain-like” (b.85.6), a widespread protein in cells (but not in viruses) that is involved in molybdopterin cofactor synthesis (Xiang et al., 2001), and (vi) the “SET domain” (b.85.7) found in both cells and viruses and involved in a range of metabolic and transport processes. The wide distribution of b.85 fold in cellular proteins (and its association with cell-related processes) suggests this fold was co-opted numerous times in evolution. Perhaps, its unique presence in some viral proteins (i.e., b.85.2 and b.85.4) could be due to convergent evolution. FSF b.85.2 however was exclusively detected in *Caudovirales* and was completely absent in Archaea and Eukarya (0%) and only detected in 8 out of 1,115 sampled bacterial proteomes (0.72%) (Table 1) suggesting it could still supplement HK97-like lineage characterization together with d.183.1 and e.48.1 FSFs. Interestingly, however, d.183.1 was highly abundant in bacterial proteomes (32%) (Table 1) indicating either widespread bacteriophage mediated gene transfer (Canchaya et al., 2003) or possibly relics of ancient co-existence of viruses in primordial cells (Nasir et al., 2012; Nasir and Caetano-Anollés, 2015). Indeed, the HK97-like lineage is the second lineage after the PRD1/Adenovirus lineage that includes viral members infecting all three cellular domains of life (Abrescia et al., 2012) suggesting it originated prior to (or concurrently with) the diversification of cellular life (Bamford, 2003; Benson et al., 2004).

### The BTV-like lineage

This lineage included three families of dsRNA viruses, *Cystoviridae, Reoviridae*, and *Totiviridae* (Abrescia et al., 2012). Members of these families encode both an outer and inner capsid core. The inner core is evolutionarily conserved and is required within the host cell to avoid apoptotic response against foreign dsRNA genomes (Grimes et al., 1998). The major core protein VP3, which forms the inner shell of the *Bluetongue virus* capsid, characterizes this lineage. About 120 monomers of VP3 are packed with icosahedral symmetry following a rather unique pattern of subunit assembly. This arrangement was also detected in the *Saccharomyces cerevisiae virus L-A* (*Totiviridae*) (Castón et al., 1997) and *Pseudomonas phage phi 6* (*Cystoviridae*) viruses (Huiskonen et al., 2006) suggesting the architecture may be unique to dsRNA viruses (Abrescia et al., 2012). VP3 is a multidomain protein containing different FSFs (Figure 1). We discovered that “A virus capsid protein alpha-helical domain” (a.115.1), “Reovirus inner layer core protein p3” (e.28.1), and “L-A virus major coat protein” (e.42.1) FSFs likely correspond to VP3-like architectures, while the “Outer capsid protein sigma 3” FSF (d.196.1) was associated with the outer core of the *Reoviridae* capsid. These FSFs were detected in the members of *Reoviridae* and *Totiviridae* (but not *Cystoviridae*). Birnaviruses, which also encode a dsRNA genome, were classified in the picornavirus-like lineage because current knowledge dictates that they exhibit stronger affinity with the “jelly-roll” fold harboring viruses (Abrescia et al., 2010). Another capsid/coat related FSF detected in *Reoviridae* is the “Viral protein domain” (b.19.1). This protein is part of capsids in *Reoviridae* (Grimes et al., 1995; Mathieu et al., 2001; VP6 and VP7 Basak et al., 1996) but is also present in minus-ssRNA (*Orthomyxoviridae*) (Rosenthal et al., 1998; Ha et al., 2002) and plus-ssRNA (members of *Nidovirales*) viruses. Structurally, the domain exhibits similarity to the “jelly-roll” fold. Thus, “jelly-roll”-like fold structures are seen in each of the four major structural lineages and also in some cellular proteins. Consistent with the signature folds of the PRD1/Adenovirus and HK97-like lineages, none of the five FSFs described here (a.115.1, e.28.1, e.42.1, d.196.1, and b.19.1) had any SCOP relatives and their presence in cellular proteomes was near negligible (Table 1). The host range of the BTV-like lineage is restricted to eukaryotic organisms (Table 2).

### Four additional candidate lineages

**Figure 3:**
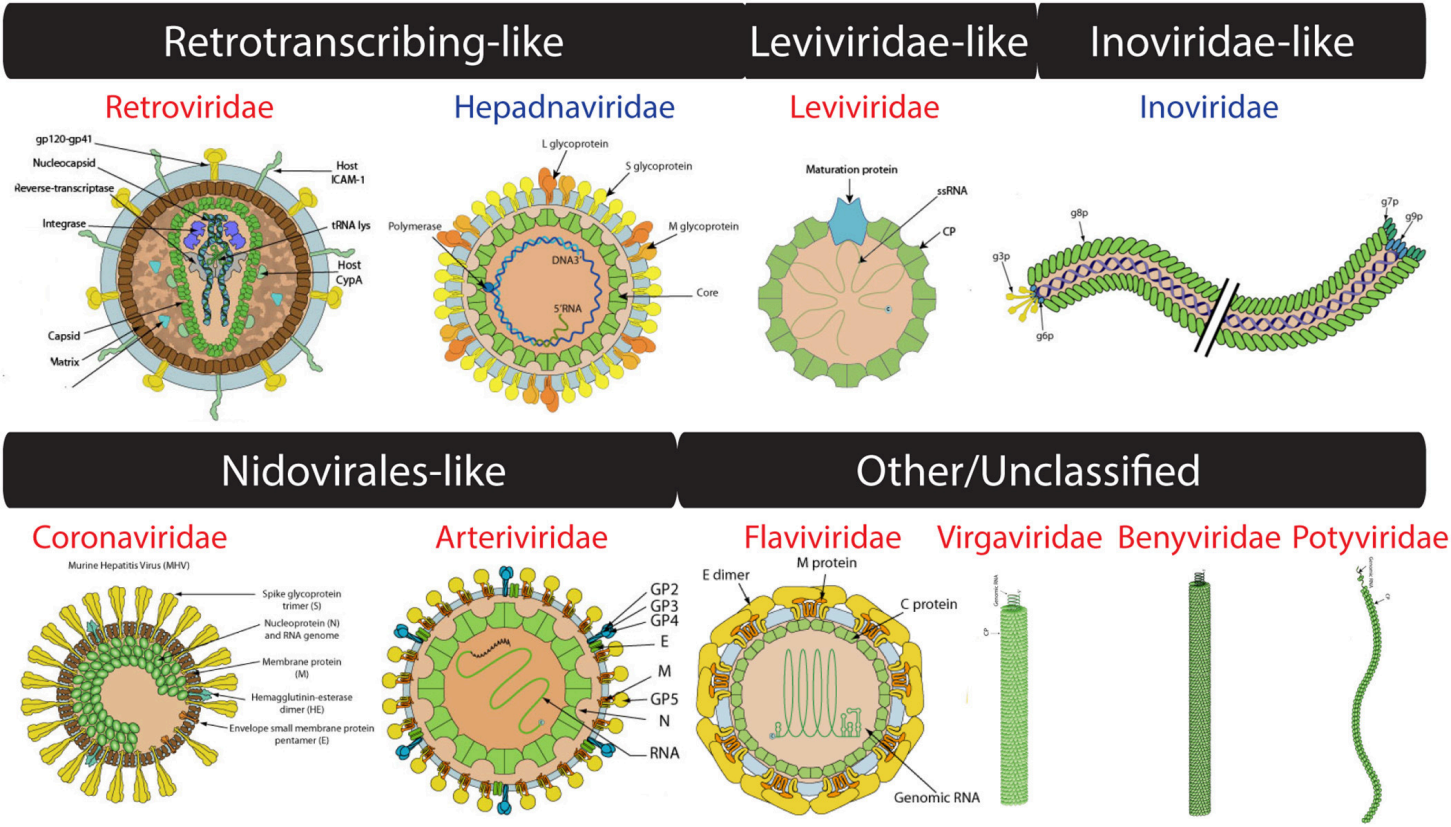
**Virion images of viral families representatives of the four computationally-defined lineages of unclassified viruses**. RNA and DNA viruses are shown in red and blue, respectively. Only RNA 1 segment shown for *Benyviridae*. Virion pictures were taken from ViralZone (Hulo et al., 2011) with permission from the Swiss Institute of Bioinformatics (SIB).

**Figure 4:**
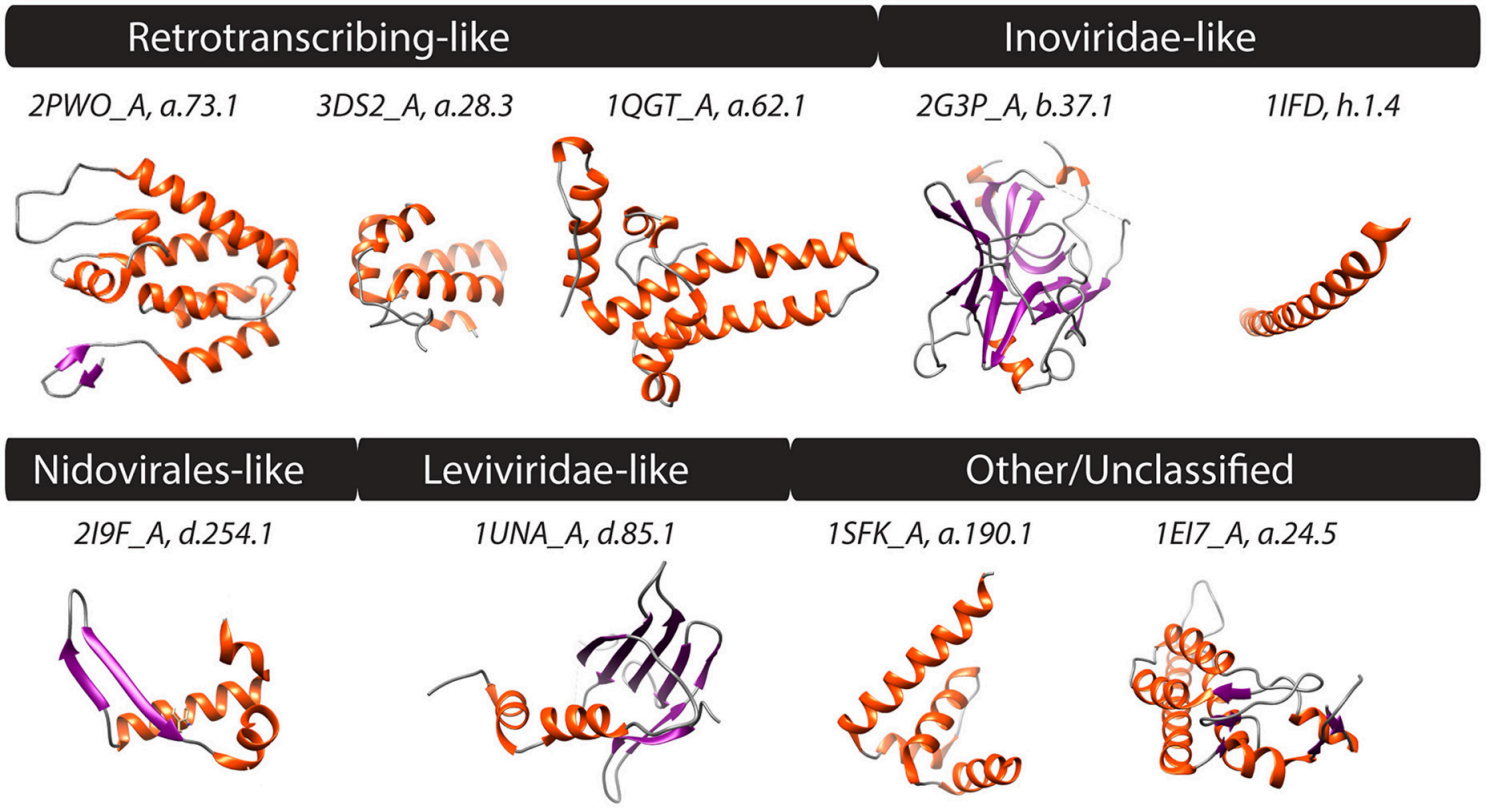
**PDB structures corresponding to FSFs of four computationally defined viral lineages along with those unclassified are shown**. Helices, strands, and coils are colored red, blue, and gray, respectively. Text above structures indicates PDB IDs along with chain information and FSF *ccs*. Synthetic structures (j.54.1 and j.9.7) not shown. Additional monomers, ligands, and extra molecules were removed from visualization. 2PWO and 3DS2, Human immunodeficiency virus type 1; 1QGT, Hepatitis B virus; 2G3P and 1IFD, Bacteriophage fd; 2I9F, Equine arteritis virus; 1UNA, Bacteriophage GA; 1SFK, Kunjin virus, and 1EI7, Tobacco mosaic virus, vulgare strain.

The ssRNA-RT (*Retroviridae*) and dsDNA-RT (*Caulimoviridae* and *Hepadnaviridae*) (Figure 3) viruses were not part of any of the four lineages in either (Abrescia et al., 2012) or our initial assignments (see above). Retrotranscribing viruses are typically enveloped and their proteins are difficult to crystalize for structural studies. The capsid protein fold from *Retroviridae* contains an N-terminal domain (5-helix bundle) involved in core formation and a C-terminal domain (4-helix bundle) involved in capsid dimerization (Jin et al., 1999; Campos-Olivas et al., 2000). These domains correspond to the “Retrovirus capsid protein, N-terminal core domain” (a.73.1) and the “Retrovirus capsid dimerization domain-like” (a.28.3) FSFs (Figure 4) and were detected in many viruses belonging to *Retroviridae* (e.g., *Human Immunodeficiency virus-1*). In contrast, the capsid fold from *Hepadnaviridae* (e.g., *Hepatitis B virus*) is also helical (5-helices) and obeys a T = 4 icosahedral symmetry. This fold corresponds to the “Hepatitis B viral capsid (hbcag)” FSF (a.62.1) (Figure 4) and was detected in members of *Hepadnaviridae*. It has been hypothesized that the C-terminal domain of HIV-1 capsid protein shows significant similarities to the HBV capsid protein suggesting that the two lineages could be evolutionarily related (Zlotnick et al., 1998). We note that the capsid fold of *Hepadnaviridae* is arranged in an array-like structure where two long helices form a hairpin that dimerizes into a 4-helical bundle closely resembling the 4-helical bundle of *Retroviridae* capsid (a.28.3). However, retroviral FSFs (a.28.3 and a.73.1) did not group with the capsid FSF from *Hepadnaviridae* (a.62.1) according to SCOP classification. Search against the DALI server (Holm and Rosenstrom, 2010) also failed to detect any apparent structural homology between the two domains (Wynne et al., 1999). Therefore, more work is required to establish if the capsids from retrotranscribing viruses form independent lineages or just one (i.e., Retrotranscribing-like lineage?). However, capsids from both *Retroviridae* and *Hepadnaviridae* are helical and this is in sharp contrast to the β-sheet rich capsids typically found in other lineages. While the *Hepadnaviridae* a.62.1 FSF was completely absent in all cellular proteomes, the two *Retroviridae* FSFs (a.28.3 and a.73.1) were exclusively detected in 17 and 5% eukaryotic proteomes but none of the prokaryotic proteomes (Table 1). Again, virus-to-cell HGT cannot be ruled out considering retrotranscribing viruses are hitherto unknown to infect prokaryotes (Nasir et al., 2014, 2015; Koonin et al., 2015). Both a.62.1 and a.73.1 had no additional SCOP relatives. FSF a.28.3 had two additional relatives: (i) “ACP-like” (a.28.1), and (ii) “Colicin E immunity proteins” (a.28.2), the former detected both in cells and viruses while the latter only in Bacteria. Because the three FSFs are unique to retrotranscribing viruses, they can serve as useful markers to fish retrotranscribing viruses from virome metagenome samples. Other enveloped viruses such as *Flaviviridae* are also hard to classify based on core capsid proteins. The virions of Flaviviruses are composed of three proteins, C, E, and M (Figure 3). The aggregation of C protein forms the nucleocapsid, which encloses the plus-ssRNA genome of flaviviruses. This protein belongs to the “Flavivirus capsid protein C” FSF (a.190.1, the sole member of the fold) that was not detected in any other family besides *Flaviviridae* or in any of the sampled cellular proteomes (Table 1, Figure 4) again indicating its reliability in characterizing viruses. However, there is indication that instead of the nucleocapsid core, surface glycoproteins involved in membrane fusion may be more similar to other enveloped viruses and could be better markers for taxonomy characterization (Abrescia et al., 2012).

In addition to the FSFs described above that were benchmarked against previous work (Abrescia et al., 2012), several other capsid/coat related FSFs unique to some viral families were also detected. For example, another candidate for novel viral lineage could be the “RNA bacteriophage capsid protein” FSF (d.85.1) (Figure 4) that was detected in several RNA viruses of bacteria (*Leviviridae*) (Figure 3). Structurally, the d.85.1 FSF is composed of 6-stranded β-sheet followed by two α-helices. It was not detected in any other viral family beside *Leviviridae* (and only in 0.26% eukaryotic proteomes) and thus could be used to characterize Leviviruses (*Leviviridae*-like lineage? also speculated to be a new lineage by Abrescia et al., 2012). Similarly, the “Nucleocapsid protein dimerization domain” FSF (d.254.1) was detected in *Coronoviridae* and *Arteriviridae*, belonging to viral order *Nidovirales* (Cavanagh, 1997) and in none of the cellular proteomes. *Coronoviridae* and *Arteriviridae* are common pathogens of animals and humans (e.g., SARS). Structurally, the domain is composed of a dimer of mixed α and β secondary structures (Figure 4). This FSF could therefore be used as bait to fish out additional members of *Nidovirales*, especially useful in quick identification of re-emergence of known coronaviruses. In turn, FSF b.37.1 represents the N-terminal domains (N1 and N2) of the gp3 minor coat protein of ssDNA bacteriophages belonging to *Inoviridae*. Structurally, the domain resembles the β-barrel fold and is primarily involved in phage infection of *E. coli*. Another domain detected exclusively in *Inoviridae* is the “Inovirus (filamentous phage) major coat protein” FSF (h.1.4), which exhibits a “pseudo-fold” comprising of oligomers of short identical α-helices (Figure 4). Together, the major and minor coat proteins (negligible presence in cellular proteomes, Table 1) can perhaps characterize inoviruses (i.e., Inovirus-like lineage?). Finally, “TMV-like viral coat proteins” FSF (a.24.5) was detected in several plus-ssRNA viruses of plants that exhibit “linear” morphology (e.g., Benyviruses, *Potyviridae*, and *Virgaviridae*). Structurally, the domain is described as a 4-helical bundle by SCOP (Figure 4). Interestingly, the major capsid proteins of archaeal linear viruses (*Lipothrixiviridae* and *Rudiviridae*) are also characterized by unique 4-helix bundles at their C-terminus. However, the arrangement of helices between *Virgaviridae* and archaeal viruses differs along with other genomic differences (Prangishvili and Krupovic, 2012; Nasir et al., 2015) suggesting perhaps that archaeal linear viruses evolved independently from bacterial and eukaryal linear viruses.

This leaves us with FSFs i.14.1, j.54.1, j.9.7, and i.7.1, for which no hits were detected in our set of sampled viral proteomes and relatively little information were available from both the SCOP and SUPERFAMILY databases (Table 1, highlighted in boldface). FSF i.14.1 is the low-resolution protein structure of “Bacteriophage HK97 procapsid (prohead II),” as defined by SCOP. Thus, it could be pooled along with other FSFs that define the HK97-like viral lineage, albeit with caution. In turn, FSF j.54.1 is the “Hepatitis C virus N-terminal capsid protein fragment 2–45.” It is a synthetic structure that is yet to be published. Whereas, j.9.7 is the “Ilarvirus coat protein N-terminal fragment” of Alfalfa mosaic virus YSMV (plus-ssRNA, *Bromoviridae*). Thus, it could be tentatively assigned to the Picornavirus-like lineage. Finally, FSF i.7.1 is defined as “Reovirus components” in SCOP. This FSF includes minor core protein lambda 3, outer capsid protein mu1, and reovirus core proteins. This could perhaps also supplement member identification of BTV-like viral lineage.

### A census for virion related proteins

As final check, we retrieved protein entries tagged to the “Virion” keyword of “Cellular component” category in UniProtKB (see Methods). These proteins were broadly defined as “*viral protein detected in the virion”* and included several capsid, envelope, matrix, and tegument proteins in addition to proteins directly involved in capsid assembly and virion formation. The list also included several proteins that are packaged into viral capsids for successful replication of viral replicon inside cells, such as the RNA-dependent-RNA polymerase of minus-ssRNA viruses and enzymes responsible for host cell membrane degradation during virus entry. These proteins therefore broadly point to an interesting set of proteins that are related to virions of viruses but not necessarily relevant for viral taxonomy. This is showcased by the fact that a total of 164 FSFs were detected in these proteins indicating the diversity and breadth of the biological process of virion synthesis (Table S1). A total of 22 (out of 23, with the exclusion of the bacteriophage procapsid FSF a.84.1) FSFs detected in our sampled proteomes (Table 1) were part of the list including FSFs b.121.4 and b.121.6 (picornavirus-like lineage), b.19.1 (BTV-like lineage), and a.73.1 and a.28.3 (retrotranscribing-like lineage?) with more than 100 hits and FSF b.121.2 (PRD1/Adenovirus-like lineage) with 92 hits (Table S1). In addition, the list included several ancient and widespread protein folds such as the P-loop containing NTP hydrolase and SAM-dependent methyltransferases, among other proteins, that were (near)-universal in cellular proteomes. In fact, 30 and 57 of these FSFs were detected in >95% prokaryotic and eukaryotic proteomes, respectively, indicating a similar use of 3D structural designs in cellular organisms, perhaps for processes other than virion synthesis or revealing a strong link to the co-existence of viral and cellular ancestors (Nasir et al., 2012, 2015; Nasir and Caetano-Anollés, 2015). Despite a significant number of mostly cellular proteins that share structural similarities to steps involved in capsid assembly and virion synthesis, the paucity of capsid-like shells in cells however remains surprising (read below).

## Discussion

Our computational approach enabled a quick scan of thousands of viral proteins against structure libraries and recovered the experimentally defined four major capsid-based viral lineages (Abrescia et al., 2012) along with proposals for new structure-based lineage additions. Only very few members were missing. This could be a result of using a stringent criterion in assigning FSFs to viral proteins (i.e., *E* < 0.0001) or alternatively absence of corresponding entries of the RCSB PDB database (Rose et al., 2015) in SCOP. Importantly, results show that viruses with different replicons and proteome histories have capsids that are structurally very similar and that HMM-based assignment (Gough et al., 2001; Gough and Chothia, 2002) reproduced the well-known viral lineages. Moreover, only a limited number of unique capsid/coat related structures (*n* = 23), mostly unique to a particular viral family or group, exist in the virosphere that can characterize viruses belonging to 4–8 known groups (Table 1). Because the discovery of unique protein folds has slowed down considerably in the past five years (e.g., 1,195 folds in SCOP 1.75 updated 2009 vs. 1,208 folds in SCOP 2.05 updated February 2015), we speculate that the 27 capsid/coat related viral protein folds identified in our study is not far from the true diversity of virion structural components in nature. The recent drive in metagenome and virome sequencing will no doubt aid in isolating new viruses harboring novel capsid/coat related folds. However, based on the observation that capsid/coat proteins repeat in viruses, we speculate that between 12 and 15 viral lineages exist in nature and the real number is likely closer to the lower bound. Remarkably, the majority of virus capsid/coat-related FSFs are either completely absent or rare in cellular organisms with exceptions likely representing virus-to-cell HGT (Nasir and Caetano-Anollés, 2015). These observations identify the capsid structure as a useful marker for defining viruses, functionally analogous and effective as 16S rRNA for the detection of prokaryotic DNA/RNA in metagenome samples.

Three limitations of the computational approach however must be noted: First, some capsid/coat protein folds characterize large groups of viruses (e.g., several plus-ssRNA virus families characterized by the “jelly-roll” fold) indicating low resolution in pinpointing the quantity and nature of viruses present in samples, while the others are unique to one family (e.g., *Leviviridae* or retro-transcribing viruses) thus indicating significant utility in recognizing specific viral groups. Thus, the quality of analysis is expected to vary from sample to sample. Second, only a qualitative assessment of viral diversity (e.g., whether retro-transcribing viruses are likely to be present in samples or not?) seems possible utilizing capsid as taxonomic marker. This is however still cheaper than either shotgun sequencing of all nucleic acids present in metagenome samples or a hybridization-capture approach of pulling down nucleic acids homologous to known viruses (Wylie et al., 2015). Both approaches are cost-prohibitive for large number of samples simply because viruses possess replicons of at least seven types and exhibit high levels of sequence polymorphisms. Third, morphological similarities in viruses can also result from convergent evolution, especially because there are only a limited number of “economical” ways to pack viral genomes. These arguments have been discussed elsewhere and were considered to be less likely (Abrescia et al., 2012). For example, in addition to sharing the same capsid fold in similar arrangement, some viral lineages also share common ATPases that package the viral genome into the capsid. Thus, additional properties favor vertical inheritance of the well-defined lineages (Abrescia et al., 2012). Moreover, protein domains grouped into common FSFs are recognized by the existence of a conserved backbone formed by unique interactions between amino acid side chains. The odds of originating the same backbone independently and multiple times in evolution are considered to be very small (0.4–4% in Gough, 2005) indicating convergence an exception and divergence the rule when evaluating similarities in structures (Abrescia et al., 2012). Nevertheless, the four new candidate structure-based lineages proposed by our study should be considered putative since vertical origin of member viruses within these new lineages remains to be established. However, because FSFs identified by our study are exclusive to viral families described, they are still invaluable markers for recognition of viral families present in unknown samples (e.g., retrovirus identification via three marker FSFs, Figure 4). Importantly, the presence of an FSF is not the sole criterion to classify a viral family into a lineage. It needs to be supported by the use of the capsid fold in similar organization and other genomic evidence (where available). Thus, it is important to consider both structural (capsid) and non-structural (polymerases and hydrolases) proteins when studying viral evolution (e.g., in Nasir and Caetano-Anollés, 2015).

Viral capsid-like architectures are relatively rare in cells. Cheng and Brooks III recently calculated distances of structural relatives of viral capsid proteins to capsid-like proteins in cells for a large number of folds (Cheng and Brooks, 2013). Using a stringent criterion (distance < 0.4), they concluded that the majority of capsid-like cellular proteins possessed variants of the “jelly-roll” fold and that these proteins were part of multi-domain proteins, which likely restricted their assembly into capsid-like structures (Cheng and Brooks, 2013). Notable exceptions however are of bacterial carboxysomes that show morphological resemblance to viral capsids but utilize folds not detected in extant viral proteomes (Yeates et al., 2007, 2011) and archaeal protein nanocompartments that store metabolic enzymes and utilize protein fold exhibiting strong homology to the HK97 fold (Sutter et al., 2008). One obvious shortcoming is the lack of classification for enveloped viruses, lack of viral representatives in the RCSB PDB database, and current biases toward sequencing economically and industrially important viruses (Delwart, 2013). These shortcomings however will naturally be overcome with the completion of ongoing and planned (meta)-genome sequencing projects. We expect that increased sequencing of novel viruses, from atypical habitats and hosts, a logical outcome of recent trends toward metagenomics and environmental sampling, can considerably bridge this gap in the near future. We therefore conclude that while the proposal of capsid structure-based viral classification seems promising, more work is required to establish boundaries within the virosphere. Remarkably, the HMM-based computational exercise impressively complements the experimental-based research and can be used to quickly determine the nature of newly discovered viruses and will aid in the qualitative assessment of viral diversity in metagenome samples.

## Author contributions

AN and GC contributed equally to this work.

## Funding

Research was supported by grants from the National Science Foundation (OISE-1132791) and the National Institute of Food and Agriculture (ILLU-802-909 and ILLU-483-625) to GCA and from the Higher Education Commission, Start-up Research Grant Program (Project No: 21-519/SRGP/R&D/HEC/2014), Pakistan to AN.

### Conflict of interest statement

The authors declare that the research was conducted in the absence of any commercial or financial relationships that could be construed as a potential conflict of interest.

## Supplementary material

Table S1 **List of 164 FSFs identified via UniProtKB keyword search**. FSFs are identified both by SCOP Ids and *ccs*. For each FSF, the total number of hits detected by HMM search and its percentage representation out of 122 archaeal (A), 1,115 bacterial (B), and 383 eukaryotic (E) proteomes are also given along with viral replicon association. FSFs matching to Table 1 (i.e., *bona fide* capsid/coat related FSFs) are highlighted.
